# Supplementary material for: Contractile perinuclear actomyosin network promotes peripheral and polar chromosome interaction with the mitotic spindle
Source: eLife. 2026 Jun 11;15:RP110952. doi: 10.7554/eLife.110952 (PMC13258745; doi:10.7554/eLife.110952)
Supplement: Peer review history

# Editor report: eLife Assessment

**DOI:** 10.7554/eLife.110952.3.sa0

**Authors:** Silke Hauf

**Affiliations:** Virginia Tech United States

**Keywords:** Convincing, Important

This **important** study demonstrates that a perinuclear actomyosin network, present in some types of human cells, facilitates kinetochore-spindle attachment of chromosomes in unfavorable locations, thereby reducing their missegregation rate. This actomyosin network and its general role have been studied previously, but this study **convincingly** clarifies the underlying mechanism and expands the investigation to additional cell lines. The results are relevant to understanding chromosome missegregation in cancer cells.

[Editors' note: this paper was reviewed by Review Commons.]

# Referee report: Reviewer #1 (Public review)

**DOI:** 10.7554/eLife.110952.3.sa1

Sheidaei and colleagues report a novel and potentially important role for an early mitotic actomyosin-based mechanism, PANEM contraction, in promoting timely congression of chromosomes located at the nuclear periphery, particularly those in polar positions. The manuscript will interest researchers studying cell division, cytoskeletal dynamics, and motor proteins. Although some data overlap with the group's prior work, the authors extend those findings by optimizing key perturbations and performing more detailed analyses of chromosome movements, which together provide a clearer mechanistic explanation. The study also builds naturally on recent ideas from other groups about how chromosome positioning influences both early and later mitotic movements.

Comments on revised version:

In the revised manuscript, organizational issues have been largely resolved. In addition, the inclusion of new experiments in additional cell lines, along with an expanded discussion that places actomyosin contractility in the broader conceptual context of other mechanisms governing chromosome movement, has significantly strengthened the manuscript.

# Referee report: Reviewer #3 (Public review)

**DOI:** 10.7554/eLife.110952.3.sa2

Sheidaei et al. report how chromosomes are favourably positioned to facilitate kinetochore-microtubule interactions during early mitosis. Studying kinetochore capture during early prophase is extremely difficult due to kinetochore crowding, but the team has taken up the challenge by classifying types of kinetochore movements, carefully marking kinetochore positions in early mitosis, and linking these to map their fate/next positions over time. The work is an excellent addition to the chromosome segregation field, as most of the literature has thus far focused on tracking kinetochores at slightly later stages of mitosis. The authors show that PANEM facilitates chromosome positioning toward the interior of the newly forming spindle, which in turn promotes chromosome congression. In the absence of PANEM, chromosomes end up in unfavourable locations and fail to form proper kinetochore-microtubule interactions. The work highlights the perinuclear actomyosin network in early mitosis (PANEM) as a key spatial and temporal element of chromosome congression, a step that precedes the segregation process.

Comments on revised version:

The authors' revisions have brought clarity to the description of movements in many of the figures. The manuscript ties a fundamental process to differences in cancer cell lines.

The work extends their published discovery that an actomyosin network forms on the cytoplasmic side of the nuclear envelope during prophase. The current manuscript explains how this network facilitates chromosome capture and congression by tracking the motions of individual kinetochores during early mitosis. The findings are broadly useful for the cell division and cytoskeletal fields.

# Author comment: Author response

**DOI:** 10.7554/eLife.110952.3.sa3

**Authors:** Nooshin Sheidaei, John K Eykelenboom, Zuojun Yue, Graeme Ball, Alexander JR Booth, Tomoyuki U Tanaka

**Affiliations:** University of Dundee Dundee United Kingdom; University of Dundee Dundee United Kingdom; University of Dundee Dundee United Kingdom; University of Dundee Dundee United Kingdom; University of Dundee Dundee United Kingdom; University of Dundee Dundee United Kingdom

The following is the authors’ response to the original reviews

**Reviewer #1 (Evidence, reproducibility and clarity):**

Summary

Sheidaei and colleagues report a novel and potentially important role for an early mitotic actomyosinbased mechanism, PANEM contraction, in promoting timely congression of chromosomes located at the nuclear periphery, particularly those in polar positions. The manuscript will interest researchers studying cell division, cytoskeletal dynamics, and motor proteins. Although some data overlap with the group's prior work, the authors extend those findings by optimizing key perturbations and performing more detailed analyses of chromosome movements, which together provide a clearer mechanistic explanation. The study also builds naturally on recent ideas from other groups about how chromosome positioning influences both early and later mitotic movements.

In its current form, however, the manuscript is not acceptable for publication. It suffers from major organizational problems, an overcrowded and confusing Results section and figures, and a lack of essential experimental controls and contextual discussion. These deficiencies make it difficult to evaluate the data and the authors' conclusions. A substantial structural revision is required to improve clarity and persuasiveness. In addition, several key control experiments and more conceptual context are needed to establish the specificity and relevance of PANEM relative to other microtubule- and actin-based mitotic mechanisms. Testing PANEM in additional cell lines or contexts would also strengthen the claim. I therefore recommend Major Revision, addressing the structural, conceptual, and experimental issues detailed below.

Major Comments

A. Structural overhaul and figure reorganization

The Results section is overly dense, lacks clear structure, and includes descriptive content that belongs in the Methods. Many figure panels should be moved to Supplementary Materials. A substantial reorganization is required to transform the manuscript into a focused, "Reports"-type article.

Move methodological and descriptive details (e.g., especially from the second Results subheading and Figure 2) to the Methods or Supplementary Materials.

In these parts, we define four phases of kinetochore motion in early mitosis. Without such a description in the main text, readers would be confused about subsequent analyses. Figure 2 is also important to show examples of how the four phases develop. Although we respect this suggestion from the reviewer, we would like to keep these parts in the main text and main figure.

Remove repetitive statements that simply restate that later phenotypes arise as consequences of delayed Phase 1 (applicable to subheadings 3 onward).

As suggested, we have removed the statement for the delayed start of Phase 2 for peripheral kinetochores in azBB-treated cells (Page 9, second paragraph). We have also simplified the statement for the delayed start of Phase 3 and Phase 4 to avoid repetition (Page 9, third paragraph; Page 10, second paragraph).

Figure 4I: This panel is currently unclear and should be drastically simplified.

Following this suggestion, we simplified Figure 4I by removing the column of ‘Start’, which is easily deduced from the ‘Duration’ results and therefore does not provide much new information.

I recommend to reorganize figures as follows:

Figure I: Keep as single figure but simplify. Figure 1D and 1E could be combined, move unnormalized SCV to supplementary materials. Same goes for 1F.

We have reorganized Figure 1, as suggested, and moved unnormalized data to supplemental materials.

New Figure 2: Combine current Figures 2A, 3A, 3C, 3D, 4C, 4F, and 4H to illustrate how PANEM contraction facilitates initial interactions of peripheral chromosomes with spindle microtubules which increases speed of congression initiation.

If we were to follow this suggestion, we would lose Figure 2B, D, Figure 3B and Figure 4A, where examples of kinetochore motions are shown in images and 3D diagrams. The new Figure would mostly consist of only graphs. Without examples of images and 3D diagrams, readers would have difficulty understanding the study. Although we respect this suggestion from the reviewer, we would like to keep Figures 2, 3 and 4, as they are (except for making Figure 4I simpler; see above).

New Figure 3: Combine current Figures 5A, 5C, 5D, 5F, 6B, 6C, and lower panels of 4H to show how

PANEM contraction repositions polar chromosomes and reduces chromosome volume in early mitosis to enable rapid initiation of congression.

If we were to follow this suggestion, we would lose Figure 5B and Figure 6A, where examples of kinetochore/chromosome dynamics are shown in images and 3D diagrams. For the same reason as above, we would like to keep Figure 5 and 6 as they are, although we respect this suggestion from the reviewer.

New Figure 4: Combine Figures 7A, 7B, 7D, 7E, 7F, expanded Supplementary Figure S7, and new data to demonstrate that PANEM actively pushes peripheral chromosomes inward which is important for efficient chromosome congression in diverse cellular contexts.

We have conducted new experiments to demonstrate the role of PANEM in diverse cellular contexts, as detailed below. We have combined the new results with the original Figure S7 to create Figure 8 in line with this suggestion.

On the other hand, in our view, combining Figure 7A-E and the extended Figure S7 would be confusing because the two parts address different topics. Although we respect this suggestion from the reviewer, we would like to keep Figure 7 and the extended Figure S7 (i.e. Figure 8) separate.

B. Specificity and redundancy of actin perturbation

To establish the specificity and relevance of PANEM, the authors should include or discuss appropriate controls:

Apply global actin inhibitors (e.g., cytochalasin D, latrunculin A) to disrupt the entire actin cytoskeleton. These perturbations strongly affect mitotic rounding and cytokinesis but only modestly influence early chromosome movements, as reported previously (Lancaster et al., 2013; Dewey et al., 2017; Koprivec et al., 2025). The minimal effect of global inhibition must be addressed when proposing a localized actomyosin mechanism. Comment if the apparent differences in this approach and one that the authors were using arises due to different cell types.

We did experiments along this line, using a dominant-negative LINC construct, in our previous study (Booth et al eLife 2019). LINC-DN should more specifically remove/reduce PANEM than the global actin inhibitors mentioned above. LINC-DN attenuated the reduction of CSV soon after NEBD and increased the number of polar chromosomes (Booth et al eLife 2019); i.e. in this regard, the outcome was similar to azBB treatment in the current study. One can expect that global actin inhibitors would also inhibit the PANEM formation and show effects similar to LINC-DN. By contrast, the indicated references reported that global actin inhibitors strongly affect mitotic rounding and cytokinesis but only modestly influence early chromosome movements, as the reviewer noted. One possibility is that such differences may have arisen from different cell types – this could be important, especially given that some cells form the PANEM and others do not (Figure 8A). A second possibility is that cytokinesis, mitotic rounding and PANEM formation may rely on actin polymerization to different extents. For example, the same concentration of global actin polymerization inhibitors may affect cytokinesis, but may still allow PANEM formation to proceed without observable effects on early chromosome movements. As suggested, we discussed this topic in the Discussion (page 16, third paragraph).

Clarify why spindle-associated actin, especially near centrosomes, as reported in prior studies using human cultured cells (Kita et al., 2019; Plessner et al., 2019; Aquino-Perez et al., 2024), was not observed in this study. The Myosin-10 and actin were also observed close to centrosomes during mitosis in *X. laevis* mitotic spindles (Woolner et al., 2008). Possible explanations include differences in fixation, probe selection, imaging methods, or cell type. Note that some actin probes (e.g., phalloidin) poorly penetrate internal actin, and certain antibodies require harsh extraction protocols. Comment on possibility that interference with a pool of Myo10 at the centrosomes is important for effects on congression.

As the reviewer implies, we cannot rule out that we could not detect actin associated with the spindle or centrosomes because of the difference in methods or cell lines between the current study and the literature mentioned by the reviewer. We have therefore moderated our claim in the Discussion that ‘we did not detect any actin network inside the nucleus, on the spindle or between chromosomes’ by adding ‘at least, using the method and the cell line in the current study’ to this statement (Page 14, second paragraph). We have also cited the three references mentioned by the reviewer in the Discussion (Page 14, second paragraph). Regarding Myosin10, azBB (blebbistatin variant) should have negligible effects on class-X myosin, including Myosin-10 (Limouze et al 2004 [PMID 15548862]). It is therefore unlikely that the effects of azBB that we observed in the current study are due to the inhibition of Myosin-10. We have cited Woolner et al 2008 and another paper and discussed this topic in the Discussion (Page 14, second paragraph).

C. Expansion of PANEM functional analysis

To strengthen the conclusions and broaden the study beyond the group's previous work, PANEM function should be tested in additional contexts (some may be considered optional but important for broader impact): [underlined by authors]

Test PANEM function in at least one additional cell line that displays PANEM to rule out cell-line-specific effects.

As suggested, we have studied the effect of PANEM contraction in cell lines other than U2OS. We have found that when PANEM contraction was inhibited, the reduction in chromosome scattering was diminished in RPE1 cells (new Figure 8B, C). Moreover, we have found that inhibition of PANEM contraction increased polar chromosomes during prometaphase/ metaphase in RPE1 and HCT116 cells (which form PANEM), but not in HeLa cells (which do not form PANEM) (new Figure 8D, E). These results suggest that the effects of PANEM contraction, originally observed in U2OS cells, are also present in other cell lines (RPE1 and HCT116) that form PANEM.

Examine higher-ploidy or binucleated cells to determine whether multiple PANEM contractions are coordinated and if PANEM contraction contributes more in cells of higher ploidies or specific nuclear morphologies.

This is an interesting suggestion, but it takes lots of time to conduct such a study, and it goes beyond the scope of this paper.

Investigate dependency on nuclear shape or lamina stiffness; test whether PANEM force transmission requires a rigid nuclear remnant.

This is an interesting suggestion, but it takes lots of time to conduct such a study, and it goes beyond the scope of this paper.

Analyze PANEM's contribution under mild microtubule perturbations that are known to induce congression problems (e.g., low-dose nocodazole).

In the current study, we found that PANEM contraction affects chromosome motions in Phase 1 and Phase 3 but not Phase 2 or Phase 4. Mild microtubule perturbation itself could affect chromosome motions in all four Phases. We do not think it would be so informative to study what additional effects the reduced PANEM contraction shows when combined with mild microtubule perturbation.

Evaluate PANEM contraction role in unsynchronized U2OS cells, where centrosome separation can occur before NEBD in a subset of cells (Koprivec et al., 2025), and in other cell types with variable spindle elongation timing.

Following this suggestion, we first investigated the timing of spindle elongation, relative to NEBD, in asynchronous U2OS cells (Figure 8 – figure supplement 3). We imaged cells every 5 min (it was difficult to reasonably observe enough mitotic cells using a shorter interval). Most of the cells showed no significant change in the spindle length (distance between two spindle poles) after (or around) NEBD [e.g. Cell 1 in A] or a mild reduction in it [e.g. Cell 2 in A]. Only a small number of cells (2-3 out of 26) showed a mild increase in the spindle length after (or around) NEBD [e.g. Cell 3 in A]. Because the spindle elongation after NEBD was rare and mild, it was difficult to address how the timing of spindle elongation affects the effect of PANEM on reducing chromosome scattering and on chromosome relocation from polar regions. We explained this result and discussed this topic in the Discussion section.

Quantify not only the percentage of affected cells after azBB but also the number of chromosomes per cell with congression defects in the current and future experiments.

It is tricky to count the number of chromosomes because they frequently overlap. Counting kinetochores is more feasible, but kinetochore signals show some non-specific background (e.g. those outside of the nucleus in prophase). We therefore quantified the chromosome volume at polar regions in azBB-treated cells (Figure 6C).

D. Conceptual integration in Introduction and Discussion

The manuscript should better situate its findings within the context of early mitotic chromosome movements:

Clearly state in the Introduction and elaborate in the Discussion that initiation of congression is coupled to biorientation (Vukušić & Tolić, 2025). This provides essential context for how PANEM-mediated nuclear volume reduction supports efficient congression of polar chromosomes.

It has been a widely accepted view in the field that chromosome congression precedes biorientation, since the publication in 2006 (Kapoor et al Science 2006). Very recently, this view has been challenged by the new publication (Vukušić & Tolić, Nat comm 2025), as indicated by this reviewer. We have mentioned this new model and discussed the new interpretation of our results based on this new model, in the Discussion (page 15; ‘It has been a widely accepted view…’).

To explain the new interpretation of our results more clearly, we have a new diagram as a supplemental figure (Figure 9 – figure supplement 1) in the revised manuscript.

Explain that PANEM is most critical for polar chromosomes because their peripheral positions are unfavorable for rapid biorientation (Barišić et al., 2014; Vukušić & Tolić, 2025).

We have included such a statement in the Discussion, as a part of the new interpretation of our results based on the new model that chromosome biorientation precedes congression (see above). We have also cited the indicated two papers.

Discuss how cell lines lacking PANEM (e.g., HeLa and others) nonetheless achieve efficient congression, and what alternative mechanisms compensate in the absence of PANEM. For example, it is well established that cells congress chromosomes after monastrol or nocodazole washout, which essentially bypasses the contribution of PANEM contraction.

Following this suggestion, we discussed three possible mechanisms that could compensate for a lack of PANEM and facilitate kinetochore-MT interaction and chromosome congression, based on previous literature (Page 17): (1) the enhanced assembly rate of spindle MTs may facilitate kinetochore-MT interactions in N-CIN+ cancer cells, (2) chromosome biorientation may precede congression more frequently to promote the congression towards the spindle midplane, and (3) the balance between CENP-E, Dynein and chromokinesin’s activities may incline to greater chromosome-arm ejection forces towards the spindle midplane.

Minor Comments

These issues are more easily addressable but will significantly improve clarity and presentation.

Introduction

Remove the reference to Figure 1A in the Introduction. The portion of Figure 1 and related text that recapitulates the authors' previous work should be incorporated into the Introduction, not the Results.

As suggested in the second sentence of this comment, we have moved most of the second paragraph of the first section of Results to Introduction (Page 4) and cited Figure 1A and 1B in Introduction. We would like to keep the reference to Figure 1A in the Introduction, because showing the PANEM images at the beginning of the manuscript would help readers’ understanding of our study. In addition, citing Figure 1A in the Introduction is more consistent with the suggestion in the second sentence of this comment.

Results (by subheading)

First subheading: When introducing the ~8-minute early mitotic interval, cite additional studies that have characterized this period: Magidson et al., 2011 (Cell); Renda et al., 2022 (Cell Reports); Koprivec et al., 2025 (bioRxiv); Vukušić & Tolić, 2025 (Nat Commun); Barišić et al., 2013 (Nat Cell Biol).

As suggested, we cited these references at the indicated part of the first section of the Results (page 5).

Second subheading: Cite key reviews and foundational research on kinetochore architecture and sequential chromosome movement during early mitosis: Mussachio & Desai, 2017 (Biology); Itoh et al., 2018 (Sci Rep); Magidson et al., 2011 (Cell); Vukušić & Tolić, 2025 (Nat Commun); Koprivec et al., 2025 (bioRxiv); Rieder & Alexander, 1990 (J Cell Biol); Skibbens et al., 1993 (J Cell Biol); Kapoor et al., 2006 (Science); Armond et al., 2015 (PLoS Comput Biol); Jaqaman et al., 2010 (J Cell Biol).

Rieder & Alexander, 1990 (J Cell Biol) and Kapoor et al., 2006 (Science) have already been cited in the second section of the Results in the original manuscript. We agree that all other references should be cited in this manuscript, and they are now cited in the Introduction and/or Discussion where they fit best (e.g. Mussachio & Desai 2017 reviews the kinetochore in general and is therefore best cited in the Introduction).

Third subheading: Clarify why some kinetochores on Figure 3A appear outside the white boundaries if these boundaries are intended to represent the nuclear envelope.

We interpret that these are background signals in the cytoplasm, which do not come from kinetochores, because (1) before NEBD, they were outside of the nucleus, and (2) after NEBD, they did not show any characteristic kinetochore motions such as those towards a spindle pole (Phase 2) and the spindle mid-plane (Phase 4). We have commented on these background signals in the legend for Figure 3A.

Fourth subheading: Note that congression speed is lower for centrally located kinetochores because they achieve biorientation more rapidly (Barišić et al., 2013, Nat Cell Biol; Vukušić & Tolić, 2025, Nat Commun).

Relevant to this comment, there was an error regarding the congression speed of central kinetochores (original Figure 4H). The congression speed of peripheral kinetochores was shown correctly, but for central kinetochores it was shown incorrectly with µm per time interval (30s) shown, rather than µm per minute. We amended this error in the revised manuscript (new Figure 4H). Based on the corrected data, the speed of congression is similar between peripheral and central kinetochores. The original Figure 3G (the speed of poleward motion for central kinetochores) had a similar error, which we have also corrected in the revised manuscript. We apologize for these errors and the confusion it may have caused.

Regarding this comment, if biorientation is achieved more rapidly for central kinetochores, Phase 3 (rather than congression speed) would be shorter for central kinetochores. Indeed, Phase 3 is slightly shorter for central kinetochores (control) than for peripheral kinetochores (control) (Figure 4C), but the difference is not statistically significant (t test; *p*\=0.21).

Fifth subheading: Cite studies on polar chromosome movements: Klaasen et al., 2022 (Nature); Koprivec et al., 2025 (bioRxiv). Clarify that Figure 5F displays only those kinetochores that initiated directed congression movements.

These two references have already been cited and discussed in this Result section of our original manuscript. However, considering this suggestion, we have discussed more about polar chromosome movements reported by Koprivec et al (page 11). Meanwhile, the reviewer is correct about Figure 5F, and we have clarified this point in the Figure 5F legend.

Sixth subheading (currently in Discussion): Move the final paragraph of the Discussion into the Results and expand it with preliminary analyses linking PANEM contraction to congression efficiency across untreated cell types or under mild nocodazole treatment.

As suggested, we have moved the final paragraph of the Discussion in the original manuscript to make a new final section in the Results in the revised manuscript. Moreover, as suggested, we have studied the outcome of inhibiting PANEM contraction in cell lines other than U2OS (Figure 8 B–E), and have described the new results to the new final section in the Results.

Discussion

1. When discussing cortical actin, cite key reviews on its presence and function during mitosis: Kunda & Baum, 2009 (Trends Cell Biol); Pollard & O'Shaughnessy, 2019 (Annu Rev Biochem); Di Pietro et al., 2016 (EMBO Rep).

As suggested, we have cited all these review papers in the Discussion (page 17), and mentioned the role of the cortical actin on the spindle orientation and positioning (Kunda & Baum, 2009; Di Pietro et al., 2016), as well as the function of the actomyosin ring on cytokinesis (Pollard & O'Shaughnessy, 2019).

Significance

Advance

This study's main strength is its novel and potentially important demonstration that contraction of PANEM, a peripheral actomyosin network that operates contracts early mitosis, contributes to the timely initiation of chromosome congression, especially for polar chromosomes. While PANEM itself was previously described by this group, this manuscript provides new mechanistic evidence, improved perturbations, and detailed chromosome tracking. To my knowledge, no prior studies have mechanistically connected this contraction to polar chromosome congression in this level of detail. The work complements dominant microtubule-centric models of chromosome congression and introduces actomyosin-based forces as a cooperating system during very early mitosis. However, the impact of the study is currently limited by major organizational issues, insufficient controls, and incomplete contextualization within existing literature. Addressing these issues will substantially improve clarity and credibility. [underlined by authors]

We have addressed the underlined criticisms as detailed above.

Audience

Primary audience of this study will be researchers working in cell division, mitosis, cytoskeleton dynamics, and motor proteins. The findings may interest also the wider cell biology community, particularly those studying chromosome segregation fidelity, spindle mechanics, and cytoskeletal crosstalk. If validated and clarified, the concept of PANEM could be integrated into textbooks and models of chromosome congression and could inform studies on mitotic errors and cancer cell mechanics.

Expertise

My expertise lies in kinetochore-microtubule interactions, spindle mechanics, chromosome congression, and mitotic signaling pathways.

**Reviewer #2 (Evidence, reproducibility and clarity):**

In this manuscript, Sheidaei et al. reported on their study of chromosome congression during the early stages of mitotic spindle assembly. Building on their previous study (ref. #15, Booth et al., Elife, 2019), they focused on the exact role of the actin-myosin-based contraction of the nuclear envelope. First, they addressed a technical issue from their previous study, finding a way to specifically impair the actomyosin contraction of the nuclear membrane without affecting the contraction of the plasma membrane. This allowed them to study the former more specifically. They then tracked individual kinetochores to reveal which were affected by nuclear membrane contraction and at what stage of displacement towards the metaphase plate. The investigation is rigorous, with all the necessary controls performed. The images are of high quality. The analyses are accurate and supported by convincing quantifications. In summary, they found that peripheral chromosomes, which are close to the nuclear membrane, are more influenced by nuclear membrane contraction than internal chromosomes. They discovered that nuclear membrane contraction primarily contributes to the initial displacement of peripheral chromosomes by moving them towards the microtubules. The microtubules then become the sole contributors to their motion towards the pole and subsequently the midplane. This step is particularly critical for the outermost chromosomes, which are located behind the spindle pole and are most likely to be missegregated.

Significance

While the conclusions are somewhat intuitive and could be considered incremental with regard to previous works, they are solid and improve our understanding of mitotic fidelity. The authors had already reported the overall role of nuclear membrane contraction in reducing chromosome missegregation in their previous study, as mentioned fairly and transparently in the text. However, the reason for this is now described in more detail with solid quantification. Overall, this is good-quality work which does not drastically change our understanding of chromosome congression, but contributes to improving it. Personally, I am surprised by the impact of such a small contraction (of around one micron) on the proper capture of chromosomes and wonder whether the signalling associated with the contraction has a local impact on microtubule dynamics. However, investigating this point is clearly beyond the scope of this study, which can be published as it is. [underlined by authors]

The suggested topic (underlined) is intriguing. However, we agree with the reviewer that it is beyond the scope of this paper. The reviewer recommends publication of our manuscript as it is.

**Reviewer #3:**

Sheidaei et al., report how chromosomes are brought to positions that facilitate kinetochore-microtubule interactions during mitosis. The study focusses on an important early step of the highly orchestrated chromosome segregation process. Studying kinetochore capture during early prophase is extremely difficult due to kinetochore crowding but the team has taken up the challenge by classifying the types of kinetochore movements, carefully marking kinetochore positions in early mitosis and linking these to map their fate/next-positions over time. The work is an excellent addition to the field as most of the literature has thus far focussed on tracking kinetochore in slightly later stages of mitosis. The authors show that the PANEM facilitates chromosome positioning towards the interior of the newly forming spindle, which in turn facilitates chromosome congression - in the absence of PANEM chromosomes end up in unfavourable locations, and they fail to form proper kinetochore-microtubule interactions. The work highlights the perinuclear actomyosin network in early mitosis (PANEM) as a key spatial and temporal element of chromosome congression which precedes the segregation process.

Major points

(1) The complexity of tracking has been managed by classifying kinetochore movements into 4 categories, considering motions towards or away from the spindle mid-plane. While this is a very creative solution in most cases, there may be some difficult phases that involve movement in both directions or no dominant direction (eg Phase3-like). It is unclear if all kinetochores go through phase1, 2, 3 and 4 in a sequential or a few deviate from this pattern. A comment on this would be helpful. Also, it may be interesting to compare those that deviate from the sequence, and ask how they recover in the presence and absence of azBB.

To respond to this comment, we would like to first clarify how we selected kinetochores for our analysis. We selected kinetochores that can be individually tracked. If kinetochore tracking was difficult (before the start of Phase 4 in control and azBB-treated cells or before observing the extended Phase 3 in azBB-treated cells) because of kinetochore crowding, we did not choose such kinetochores. For example, related to the next comment of this Reviewer, we did not include kinetochores close to spindle poles (within 4 µm) at NEBD in our analysis for the following two reasons: First, these kinetochores often did not show clear and rapid movements towards a spindle pole, which we used to define Phase 2. Second, although we referred to kinetochore co-localization with a microtubule signal for the start of Phase 2, this was difficult for kinetochores close to spindle poles because of a high density of microtubules. As requested, we have added this comment to the Method section (page 25).

With the above selection, all selected kinetochores without azBB treatment (control) showed the poleward motion (Phase 2) and congression (Phase 4) in this order, though their extents were varied among kinetochores. All selected kinetochores with azBB treatment also showed the poleward motion (Phase 2), and some of them showed congression (Phase 4) after Phase 2. Then, Phase 1 and Phase 3 were defined as intervals between NEBD and Phase 2 and between Phase 2 and Phase 4, respectively. If no Phase 4 was observed with azBB, we judged that Phase 3 continued till the end of tracking. We have added this comment to the Method section (page 25-26).

(2) Would peripheral kinetochore close to poles behave differently compared to peripheral kinetochore close to the midplane (figure S4)? In figure 3D, are they separated? If not, would it look different?

Since we did not include kinetochores close to spindle poles (at NEBD), for which it was difficult to define Phase 2 (see our response to the above major point 1), in our analysis, the suggested comparison is not feasible.

(3) Uncongressed polar chromosomes (eg., CENPE inhibited cells) are known to promote tumbling of the spindle. In figure 5B with polar chromosomes, it will be helpful to indicate how the authors decouple spindle pole movements from individual kinetochore movements.

In contrast to CENPE-inhibited cells, azBB-treated cells did not show much tumbling of the spindle, though both cells showed uncongressed polar chromosomes. The reason for this difference may be fewer uncongressed polar chromosomes in azBB-treated cells. There were still modest spindle motions in azBB-treated cells. However, because kinetochore motions were assessed relative to a spindle pole (and other reference points on the spindle) in our study (Figure 2A, C), the modest spindle motions were offset in our analyses of kinetochore motions. We have clarified the underlined part in the Method section (page 24).

(4) The work has high quality manual tracking of objects in early mitosis- if this would be made available to the field, it can help build AI models for tracking. The authors could consider depositing the tracking data and increasing the impact of their work.

As suggested, we have included kinetochore tracking data as supplemental data in the revised manuscript (Figure 3 – source data 1–4; Figure 5 – source data 1, 2).

Minor points

(1) It will be helpful for readers to see how many kinetochores/cell were considered in the tracking studies. Figure legends show kinetochore numbers but not cell numbers.

As suggested, we have now mentioned the number of cells, where the kinetochore motions were analyzed, in the legends for Figures 3, 4, 5, and supplemental figures.

(2) Discussion point: If cells had not separated their centrosomes before NEBD, would PANEM still be effective? Perhaps the cancer cell lines or examples as shown in Figure 6A have some clues here.

Following this suggestion, we first investigated the timing of spindle elongation, relative to NEBD, in asynchronous U2OS cells (Figure 8 – figure supplement 3). We imaged cells every 5 min (it was difficult to reasonably observe enough mitotic cells using a shorter interval). Most of the cells showed no significant change in the spindle length (distance between two spindle poles) after (or around) NEBD [e.g. Cell 1 in A] or a mild reduction in it [e.g. Cell 2 in A]. Only a small number of cells (2-3 out of 26) showed a mild increase in the spindle length after (or around) NEBD [e.g. Cell 3 in A]. Because the spindle elongation after NEBD was rare and mild, it was difficult to address how the timing of spindle elongation affects the effect of PANEM on reducing chromosome scattering and on chromosome relocation from polar regions. We explained this result and discussed this topic in the Discussion section.

(3) Figure 7 cartoon shows misalignment leading to missegregation. It may be useful to consider this in the context of the centrosome directed kinetochore movements via pivoting microtubules. Is this process blocked in azBB-treated cells?

We understand that the Reviewer refers to the kinetochore pivoting mechanism around a spindle pole, which was recently reported by the Tolic group (Koprivec et al., 2026). Such a pivoting mechanism would work only when the spindle elongates (i.e. the distance between spindle poles is enlarged) after NEBD. Therefore, to address this Reviewer’s question, we tried to assess how PANEM contraction contributes to relocating polar chromosomes when the spindle elongates before or after NEBD in asynchronous U2OS cells (i.e. in the situation where the kinetochore pivoting mechanism is applied or not), as we noted above in response to Point 2. However, spindle elongation after NEBD was rare and mild, and we were unable to address this issue (see our response to Point 2). We discussed this matter in the Discussion section.

(4) Are all the N-CIN- lines with PANEM highly sensitive to azBB? In other words, is PANEM essential for normal congression in some of these lines.

Because blebbistatin could kill cells by inhibiting cytokinesis, the blebbistatin sensitivity of cell growth may not necessarily reflect how essential the PANEM contraction is for chromosome congression.

Instead, we addressed more directly how essential the PANEM contraction is for chromosome congression. We analyzed chromosome congression in RPE1 and HCT116 cells (both are NCIN-) in the presence and absence of pnBB, the inhibitor of PANEM contraction (new Figure 8D, E). With pnBB, these cells showed congression defects, suggesting that the PANEM contraction is essential for chromosome congression in these N-CIN- cells.

(5) Are congression times delayed in lines that naturally lack PANEM?

For example, it takes 10-20 min for HeLa cells (lacking PANEM) to complete chromosome congression after the NEBD (Bancroft et al 2025: https://doi.org/10.1242/jcs.163659). This is not significantly different from the time (8-18 min) for chromosome congression we observed in U2OS cells (which form PANEM). We assume that cells lacking PANEM have developed a compensatory mechanism for efficient chromosome congression – we have discussed possible compensatory mechanisms in the last paragraph of the Discussion (page 17).

(6) Page 23 "we first identified the end of congression" how does this relate to kinetochore oscillations that move kinetochores away from the metaphase plate?

The start of kinetochore oscillation was defined as the end of Phase 4 if we could track the kinetochore until that point. In some cases where the kinetochore became close to the midplane (< 2.5 µm), it was not possible to track it further due to kinetochore crowding around the spindle mid-plane – in such cases, the end of Phase 4 was assigned as the end of tracking. These definitions were not necessarily clear in the original manuscript. Moreover, in the original manuscript, it was not clearly stated that the end of Phase 4 was defined in the same way for both non-polar and polar kinetochores. We have now clarified these points in the Method section (page 25).

(7) Are spindle pole distances (spindle sizes) different in early and late mitotic cells (4min vs 6min after NEBD) in control vs azBB-treated cells? Please comment on Figure S2E (mean distance) in the context of when phase 4 is completed. Does spindle size return to normal after congression?

In Figure S2E (Figure 1 – figure supplement 6 in the revised manuscript), we did not observe a significant difference in the spindle-pole distance (the spindle size) between control and azBBtreated cells at any individual time points. The smallest p-value was 0.094 at 6.0 min. As suggested, we have explained this in the legend for this supplementary figure. Completion of Phase 4 is highly variable across different kinetochores within the same cell; thus, a general comment on its completion timing in cells is not feasible.

Significance:

The current work builds upon their previous work, in which the authors demonstrated that an actomyosin network forms on the cytoplasmic side of the nuclear envelope during prophase. This work explains how the network facilitates chromosome capture and congression by tracking motions of individual kinetochores during early mitosis. The findings can be broadly useful for cell division and the cytoskeletal fields.
